# Supplementary material for: A Synthetic Peptide CTL Vaccine Targeting Nucleocapsid Confers Protection from SARS-CoV-2 Challenge in Rhesus Macaques
Source: Vaccines (Basel). 2021 May 18;9(5):520. doi: 10.3390/vaccines9050520 (PMC8158516; doi:10.3390/vaccines9050520)
Supplement: Supplementary file 1 [file vaccines-09-00520-s001.zip › vaccines-1215170-supplementary/vaccines-1215170-supplementary.pdf]

vaccines

MDPI

Supplemental Materials

Figure S1

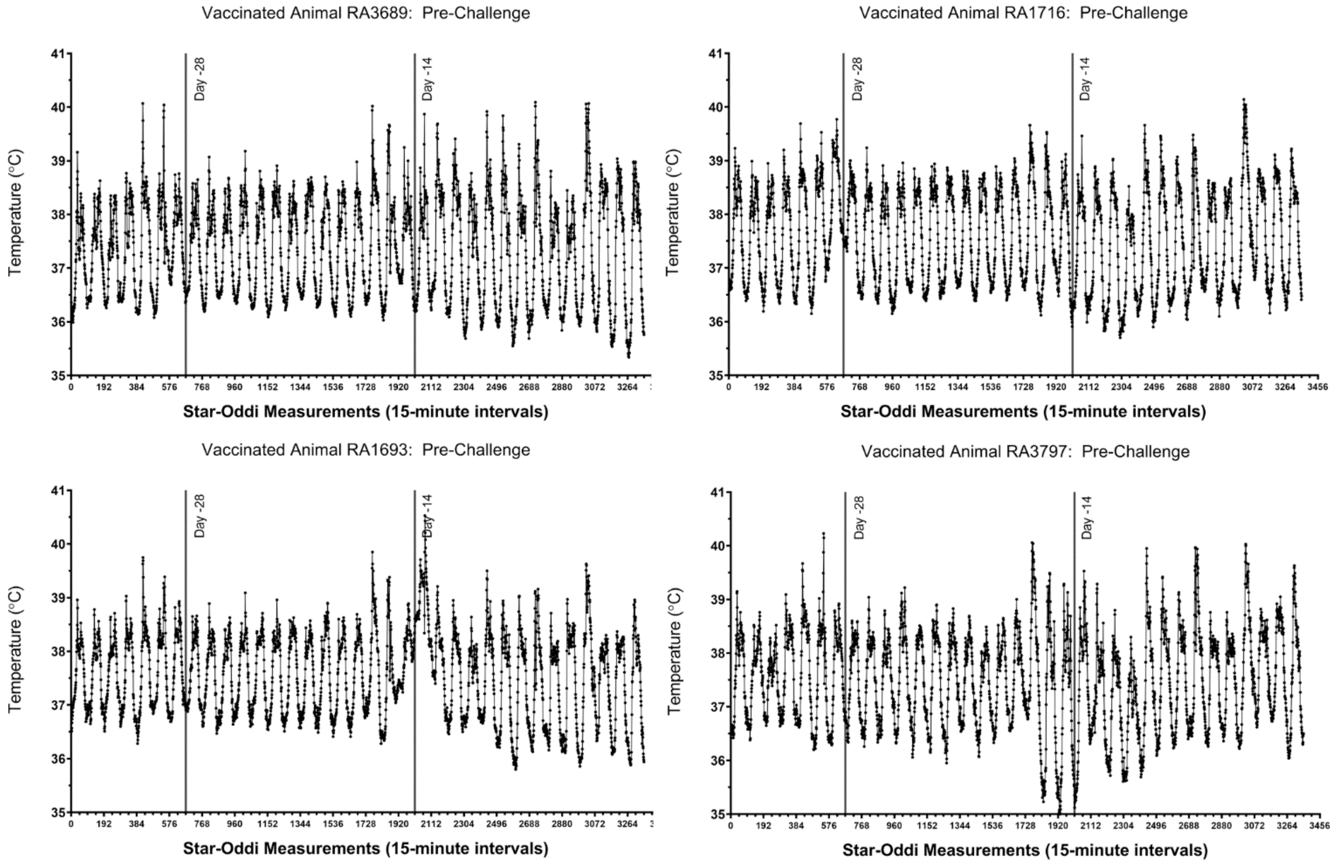

Supplemental Data, Figure S1 legend. Core body temperature alterations in vaccinated macaques prior to SARS-CoV-2 challenge. For each animal, 35 days of pre-challenge temperature measurements are shown. Each tick on the x-axis represents 12 hours or 48 individual logger measurements.

Figure S2

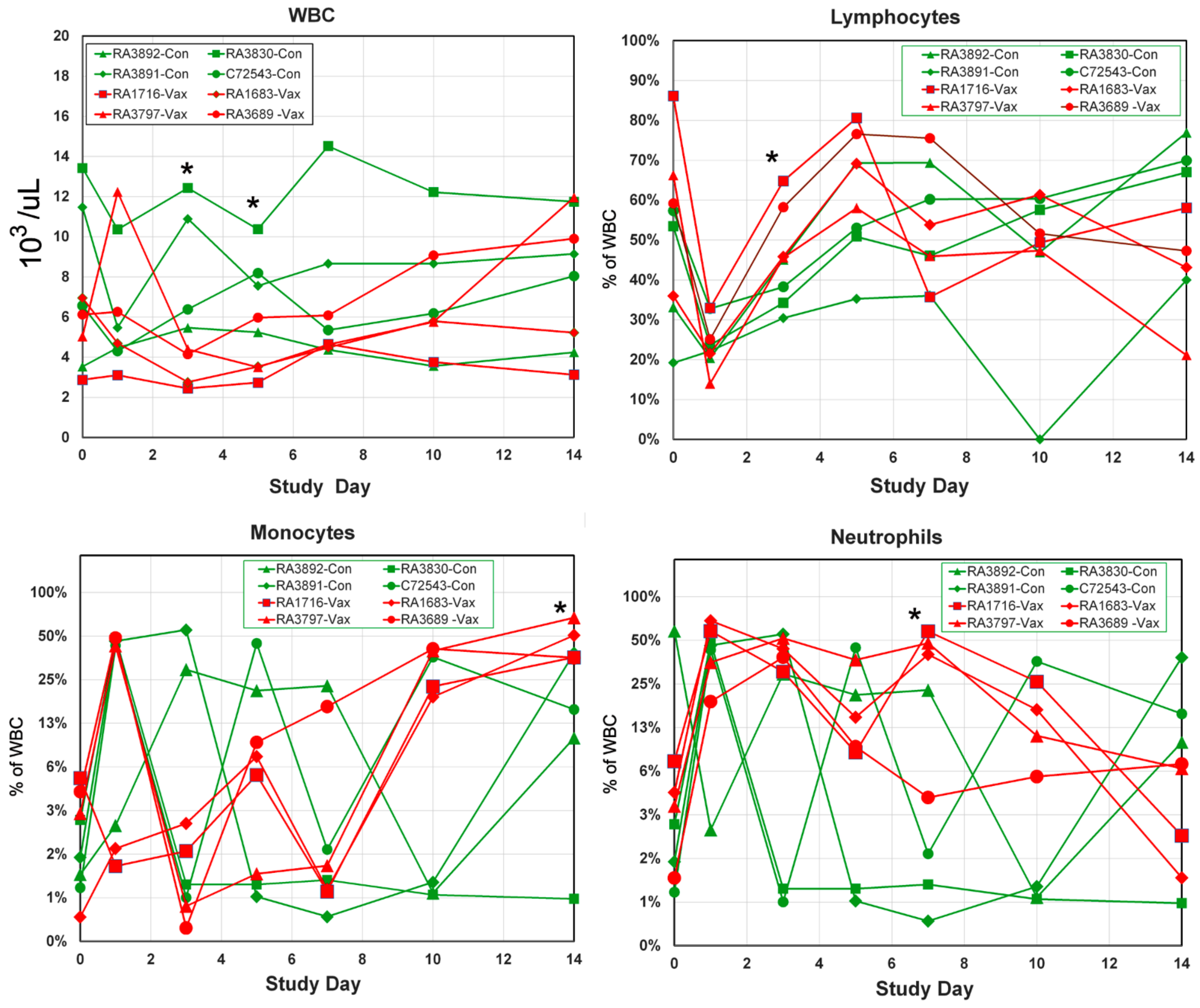

Supplemental Data, Figure S2 Legend. Hematological analysis in control and vaccinated macaques challenged with SARS-CoV-2. The counts of white blood cells (WBC) (upper left panel), the percent of lymphocytes in WBC (upper right panel), the percent of monocytes in the WBC (lower left panel), and the percent of neutrophils in the WBC (lower right panel) were analyzed. An asterisk indicates a statistically significant difference ($p < 0.05$) between control and vaccinated macaques by Students t-test.

Figure S3

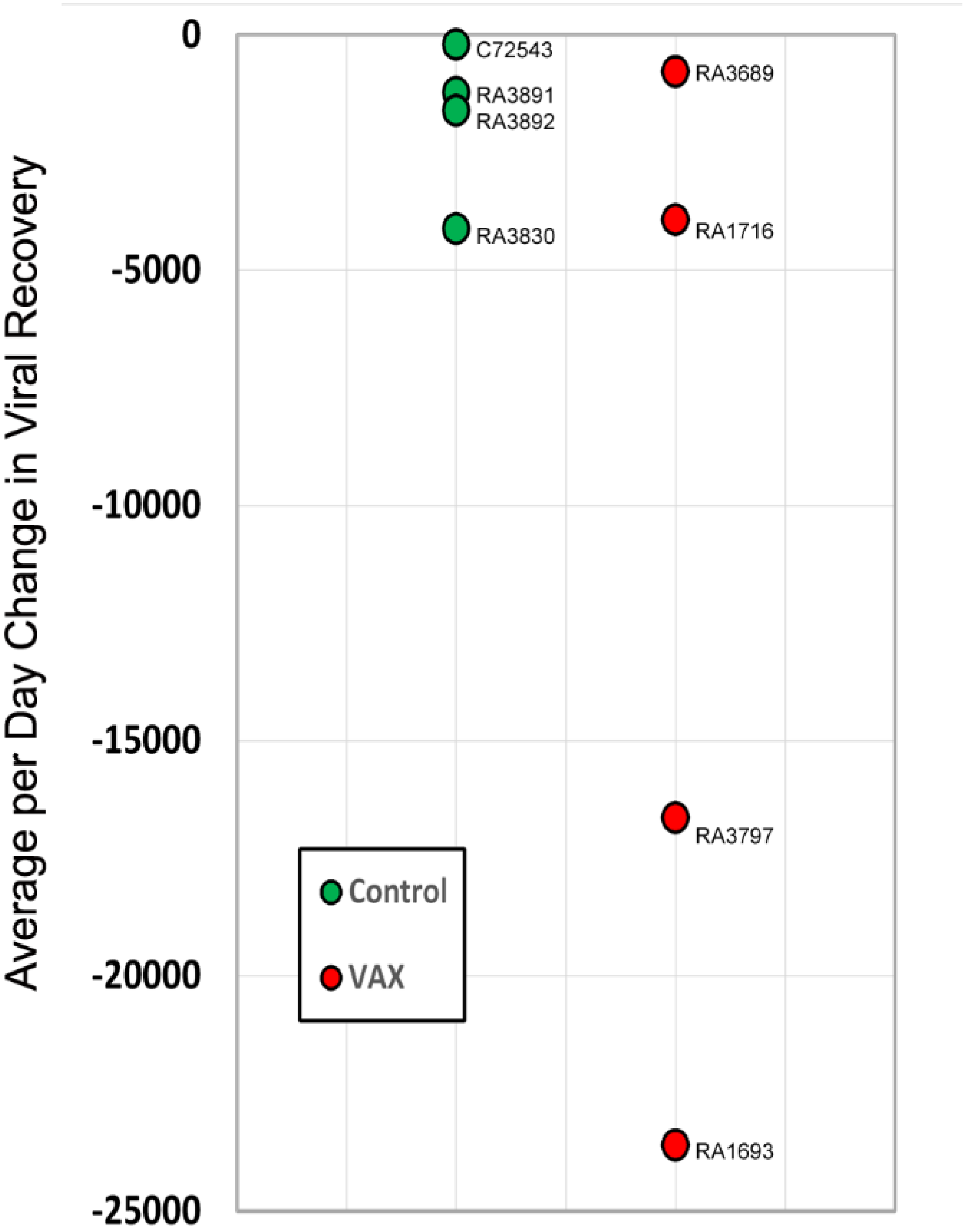

Supplemental Data, Figure S3 Legend. Viral clearance rates in control and vaccinated SARS CoV-2 challenged macaques. From the total viral loads measurements in nasal swab samples from SARS-CoV-2 challenged macaques, the daily viral clearance rates (i.e. TCID $_{50}$/mL $_{\text{day n-1}}$ minus TCID $_{50}$/mL $_{\text{day n}}$) were calculated and averaged over a nine-day period. Red symbols are vaccinated macaques subjects, control unvaccinated macaque subjects are shown in green symbols.

Figure S4

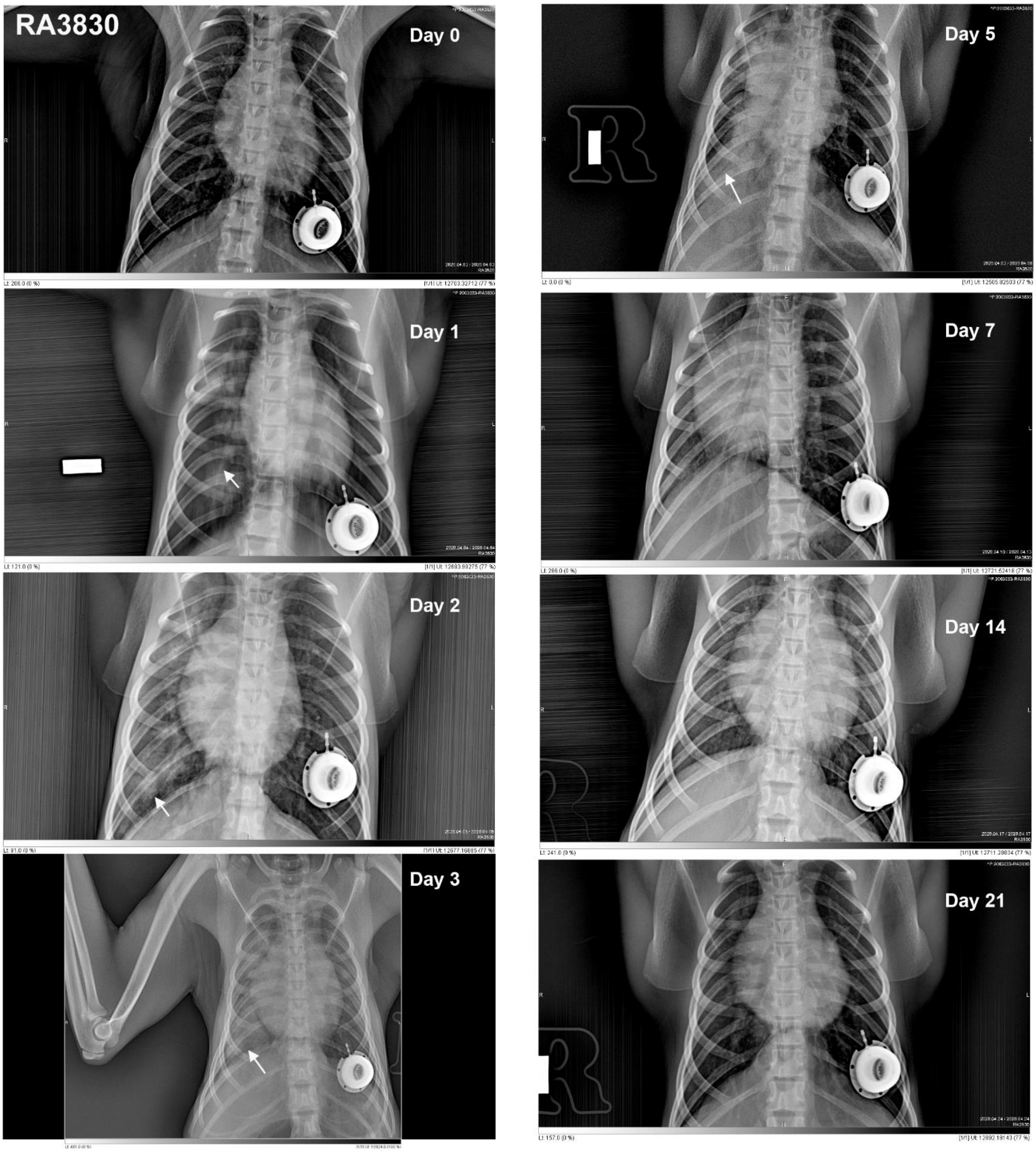

Supplemental Data, Figure S4 legend. Chest radiographs of control rhesus macaque RA3830 following SARS-CoV-2 challenge. As shown, this animal demonstrated a progression of pulmonary infiltrates during the acute period (Days 2-5) of disease post-challenge which resolved by study termination (Day 21). White arrows indicate areas of mild to moderate pulmonary infiltrates seen as ground glass consolidations.

Figure S5

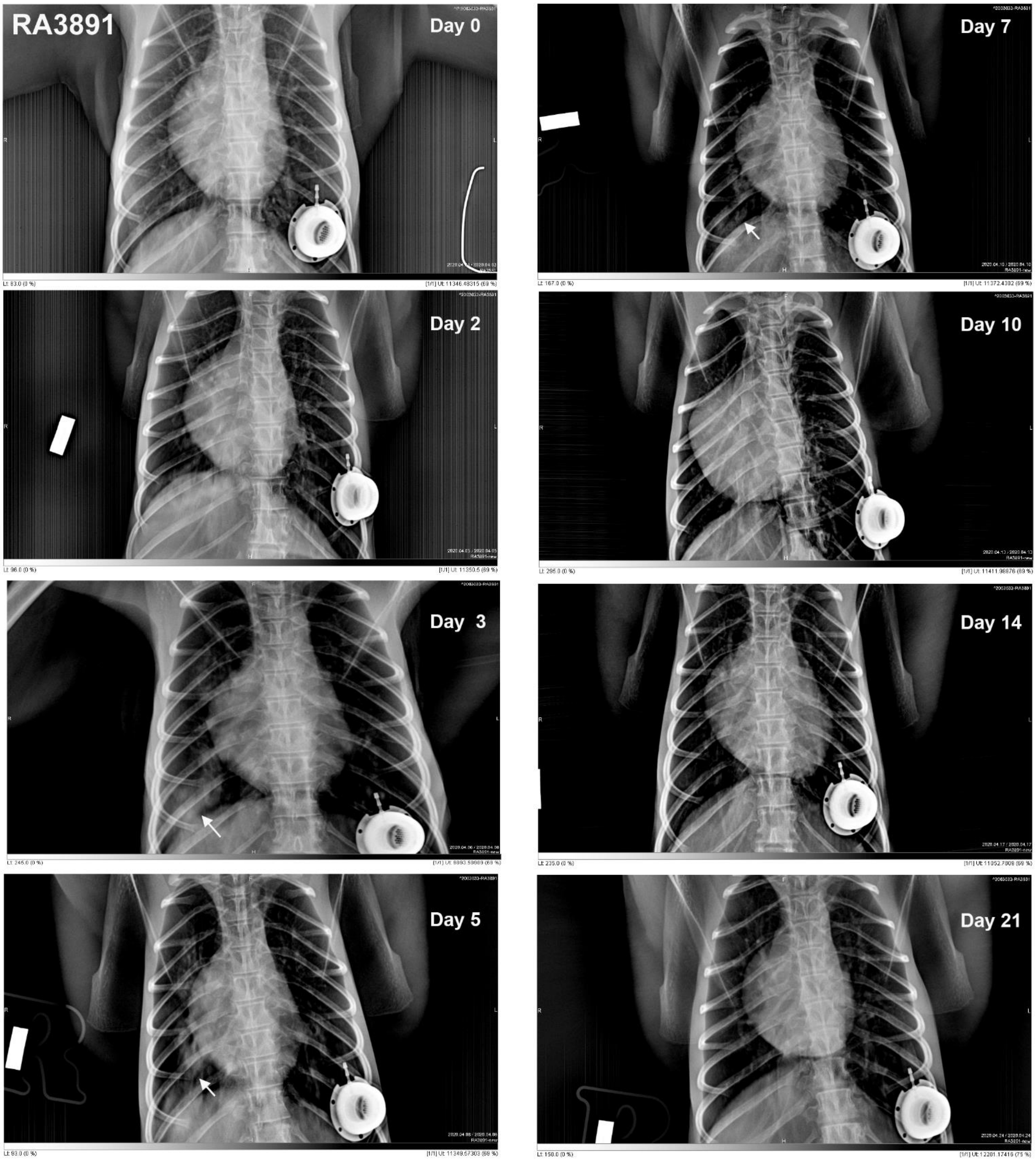

Supplemental Data, Figure S5 Legend. Chest radiographs of control rhesus macaque RA3891 following SARS-CoV-2 challenge. As shown, this animal demonstrated a progression of pulmonary infiltrates during the acute period (Days 3-7) of disease post-challenge which resolved by study termination (Day 21). White arrows indicate areas of mild to moderate pulmonary infiltrates seen as ground glass consolidations.

Figure S6

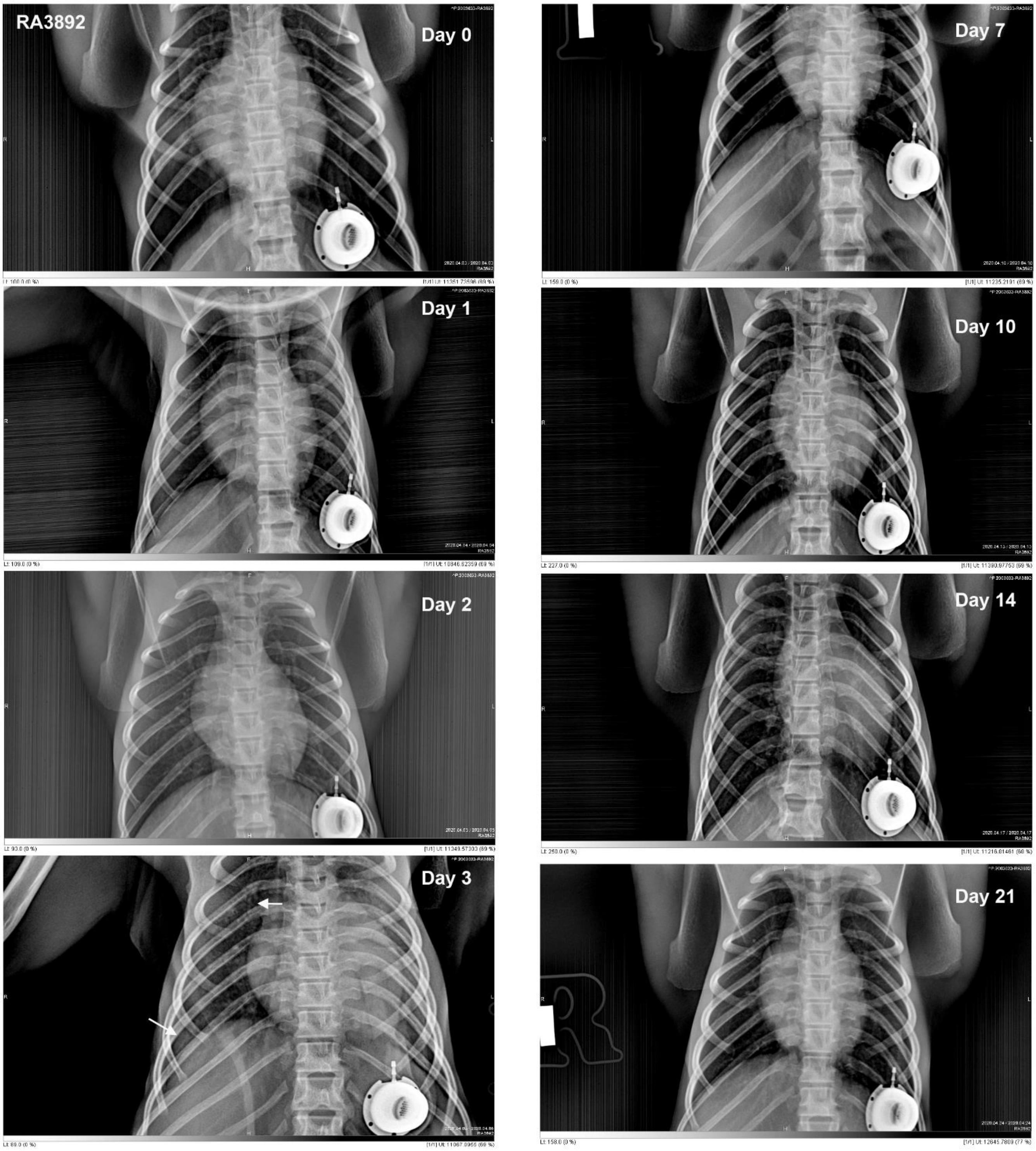

Supplemental Data, Figure S6 Legend. Chest radiographs of control rhesus macaque RA3892 following SARS-CoV-2 challenge. As shown, this animal demonstrated a progression of pulmonary infiltrates during the acute period (Day 3) of disease post-challenge which resolved by study termination (Day 21). White arrows indicate areas of mild to moderate pulmonary infiltrates seen as ground glass consolidations.

Figure S7

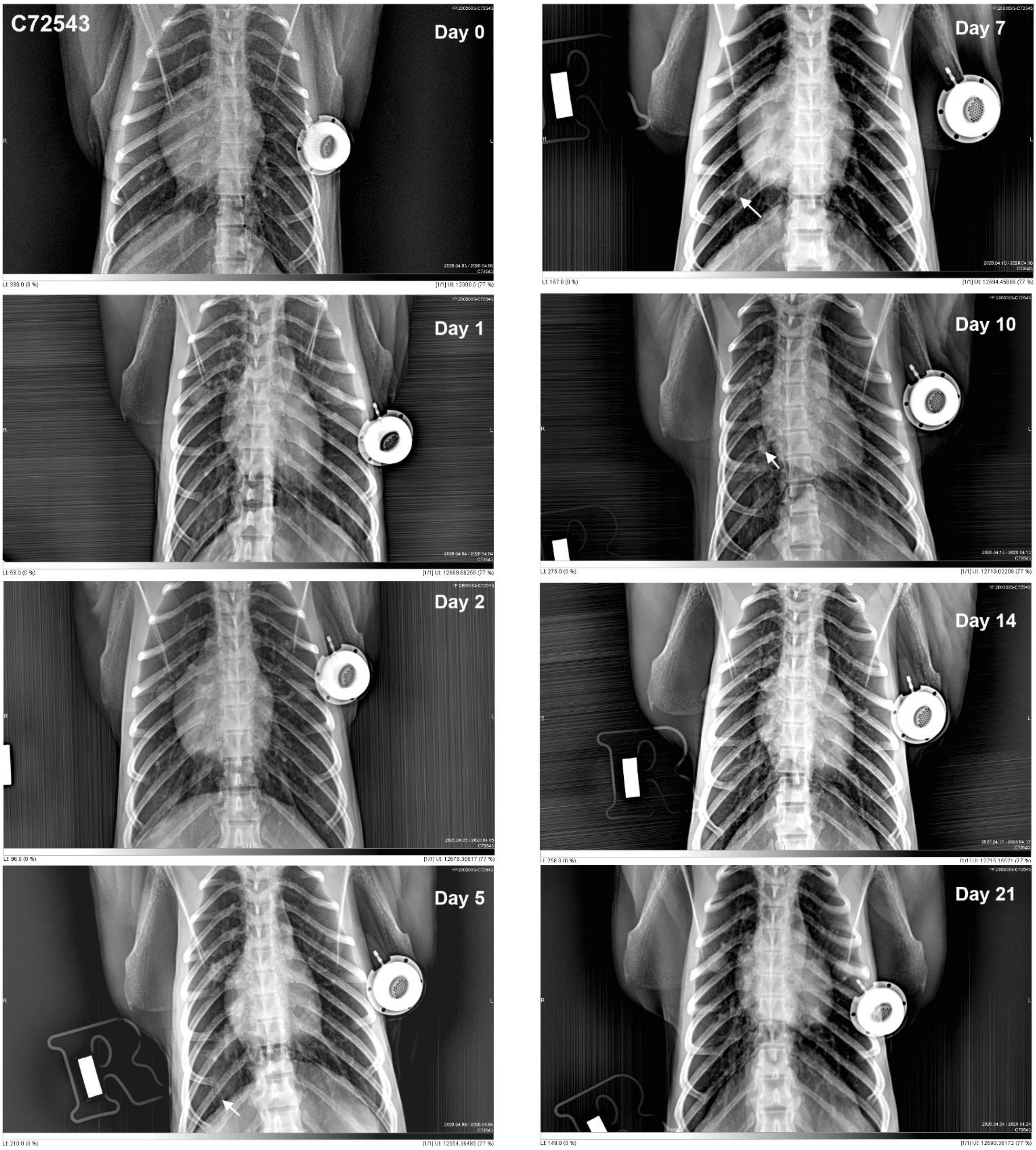

Supplemental Data, Figure S7 Legend. Chest radiographs of control cynomolgus macaque C72543 following SARS-CoV-2 challenge. As shown, this animal demonstrated a progression of pulmonary infiltrates 5-10 days post-challenge which resolved by study termination (Day 21). White arrows indicate areas of mild to moderate pulmonary infiltrates seen as ground glass consolidations.

Figure S8

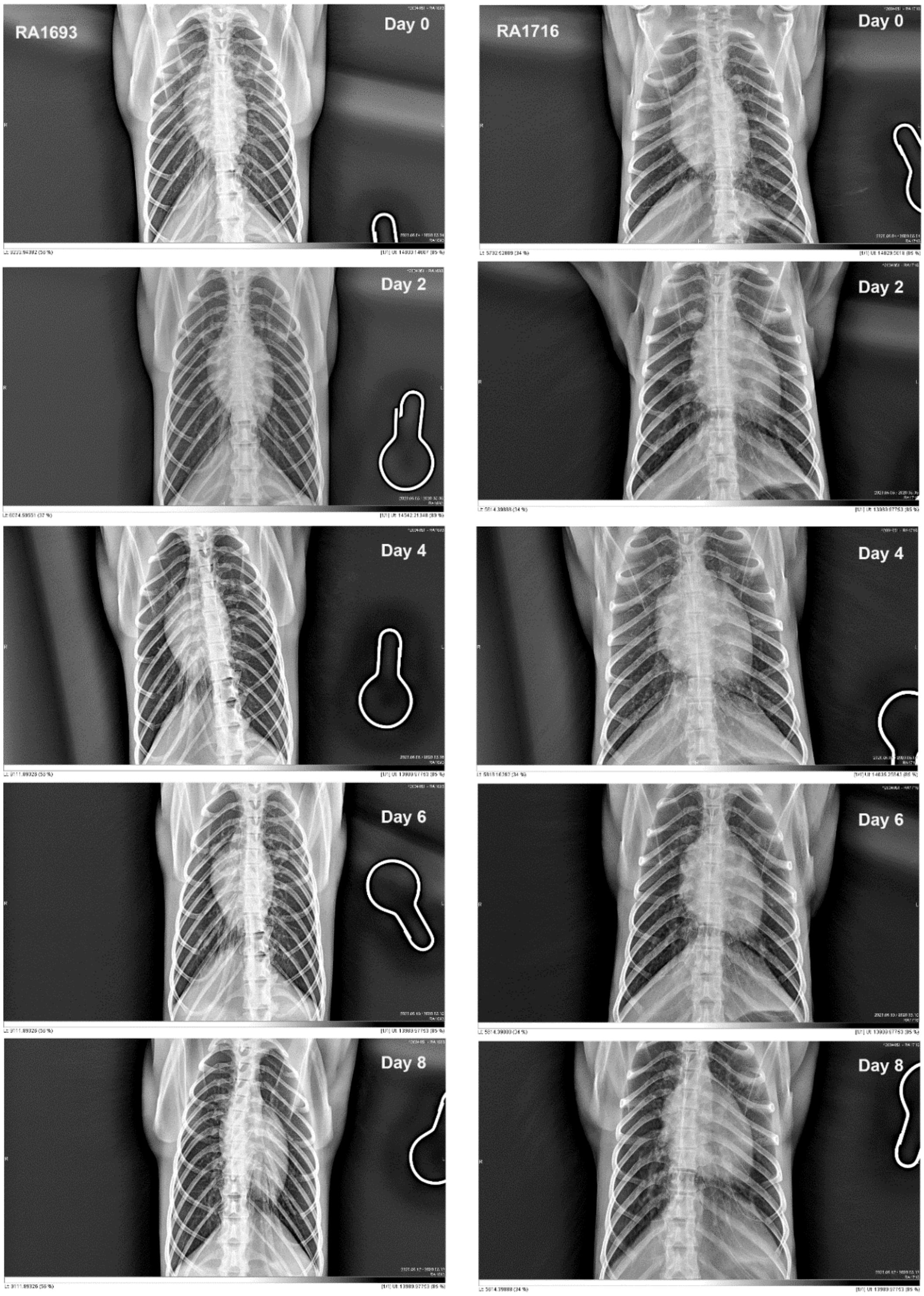

Supplemental Data, Figure S8 Legend. Chest radiographs of vaccinated rhesus macaques RA1693 and RA1716 following SARS-CoV-2 challenge. With the exception of increased reticulation relative to baseline, few abnormalities were observed in collected radiograph images. Note the absence of infiltrates or consolidation typically seen in the unvaccinated control population.

Figure S9

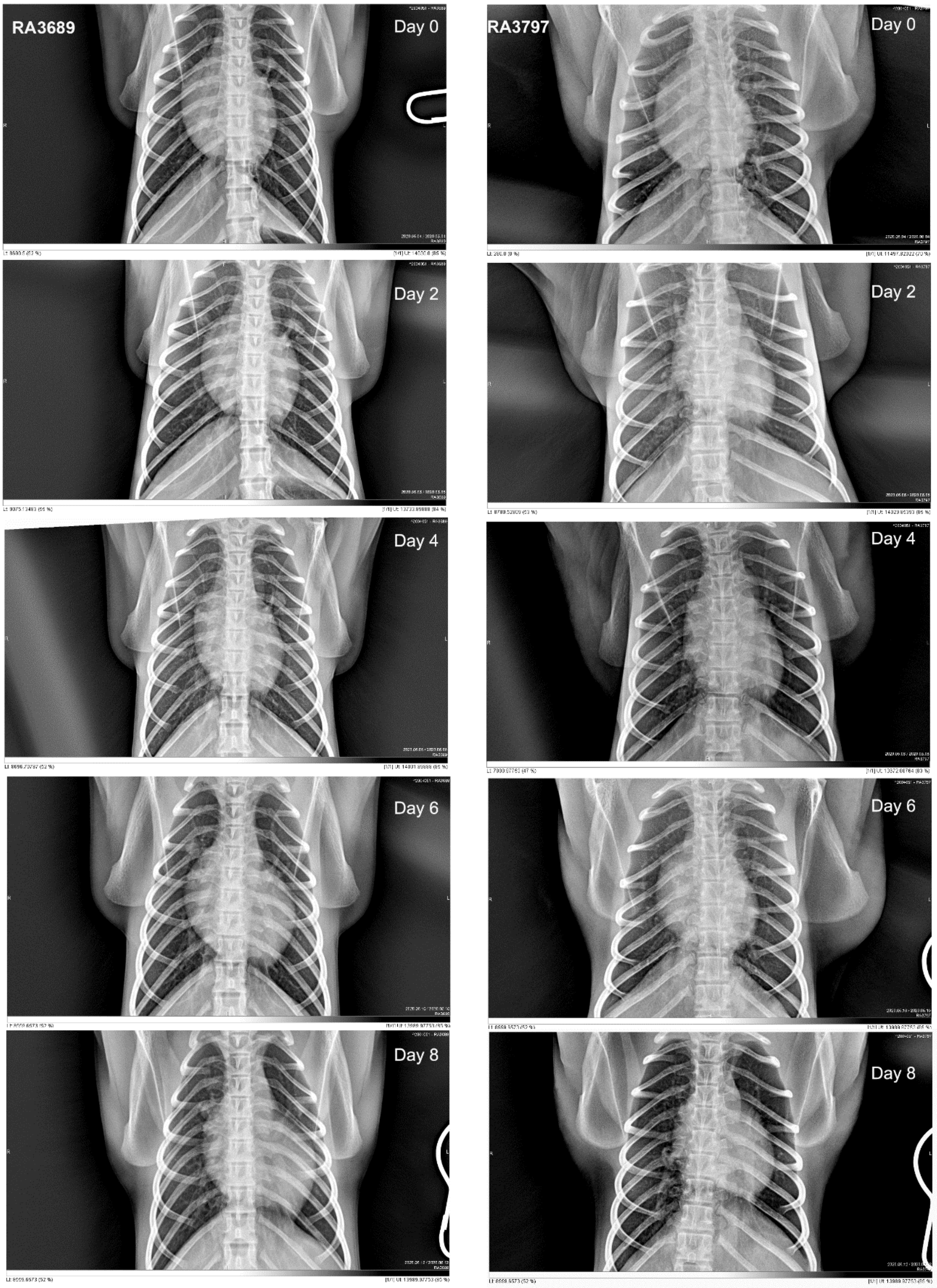

Supplemental Data, Figure S9 Legend. Chest radiographs of vaccinated rhesus macaques RA3689 and RA3797 following SARS-CoV-2 challenge. Radiographs are unremarkable, other than showing increased reticulation relative to baseline, appearing on days 2 through 4, clearing on later imaging. In particular, note lack of focal infiltrates or consolidations.

Figure S10

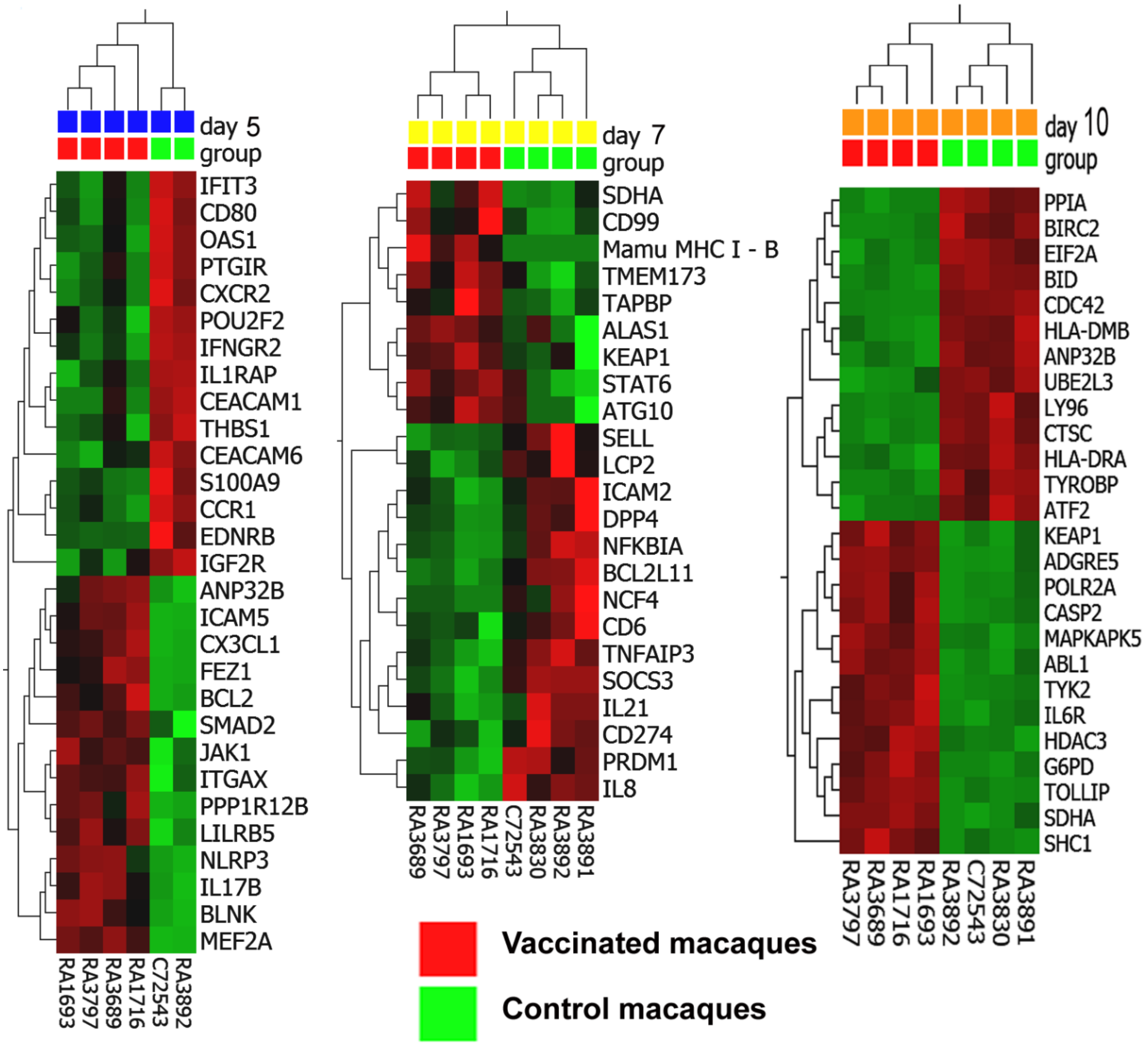

Supplemental Data, Figure S10. Hierarchical clustering of gene expression in BAL samples collected from control and vaccinated macaques on Day 5 (left panel), Day 7 (middle panel), and Day 10 (right panel). 5- and 7- days post SARS-CoV-2 challenge. Heatmap shows significantly ($p<0.05$) up-regulated (red) transcripts and down-regulated (green) transcripts from a total of 730 genes analyzed using the NanoString Non-Human Primate Immunology V2 Panel.

Figure S11

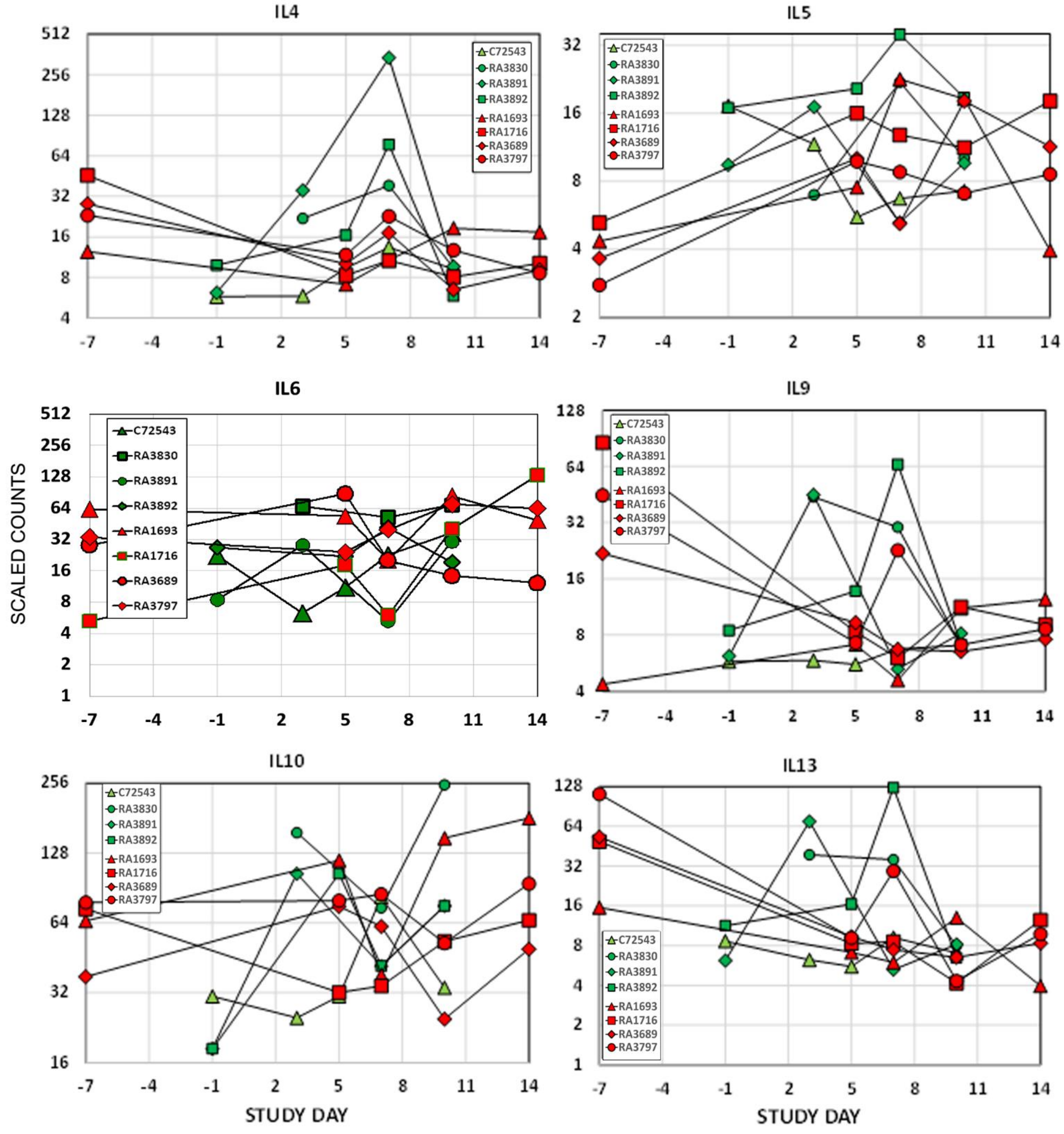

Supplemental Data, Figure S11 Legend. Intratracheal vaccination with the adjuvanted microsphere peptide vaccine did not promote the expression of Th_2 type interleukin cytokine transcripts in collected BAL samples relative to levels measured in control macaques. BAL cell gene expression (shown as scaled counts on the y-axis) of cytokines associated with Th_2 T cell responses were plotted by study day (x-axis) for each animal prior to and following the SARS-CoV-2 challenge. No statistically significant differences between control and vaccinated macaques were found.

Figure S12

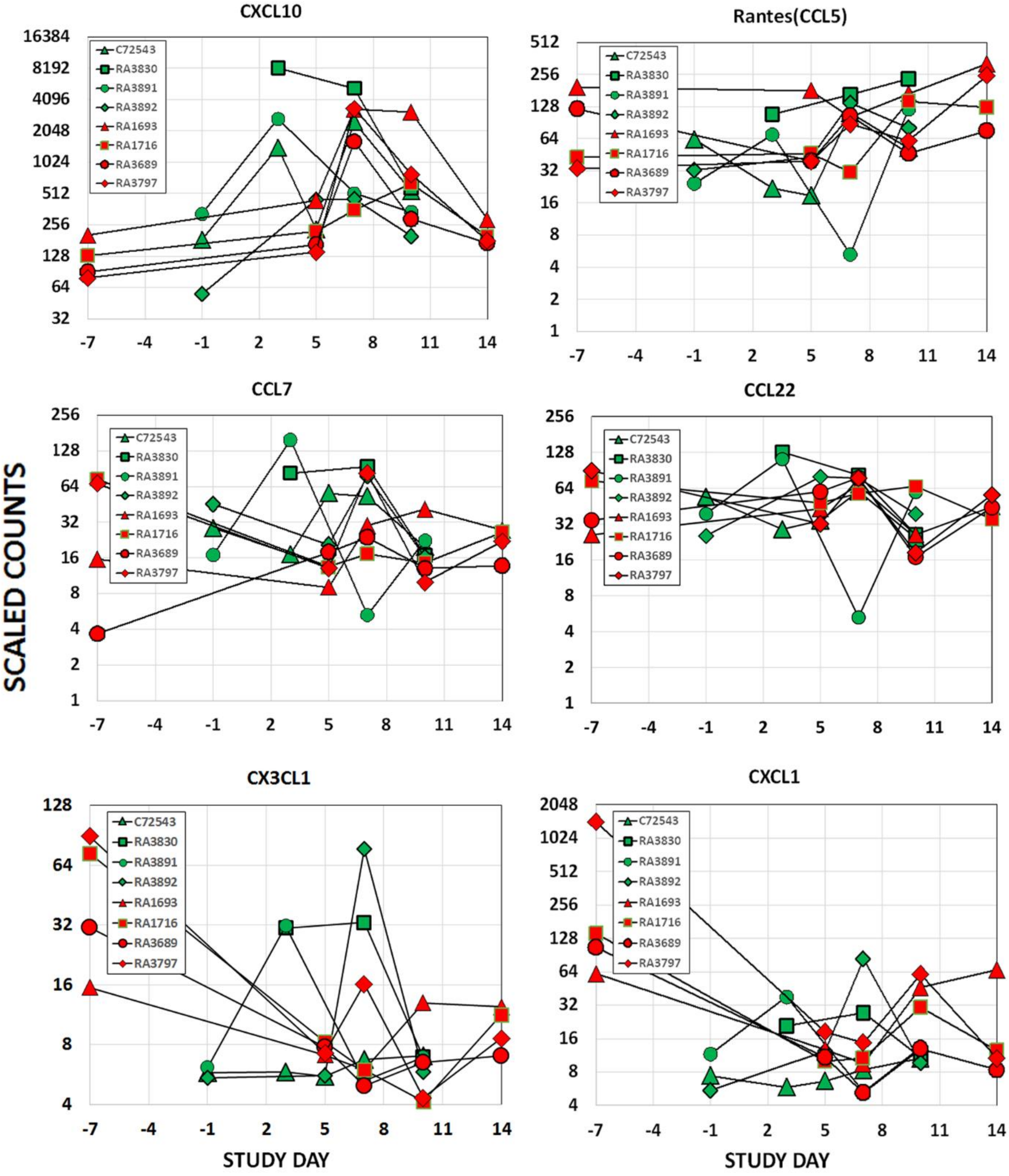

Supplemental Data, Figure S12. Intratracheal vaccination with the adjuvanted microsphere peptide vaccine did not promote the expression of Th_2 type interleukin cytokine transcripts in collected BAL samples relative to levels measured in control macaques. BAL cell gene expression (shown as scaled counts on the y-axis) of cytokines associated with Th_2 T cell responses were plotted by study day (x-axis) for each animal prior to and following the SARS-CoV-2 challenge. No statistically significant differences between control and vaccinated macaques were found.

Figure S13.

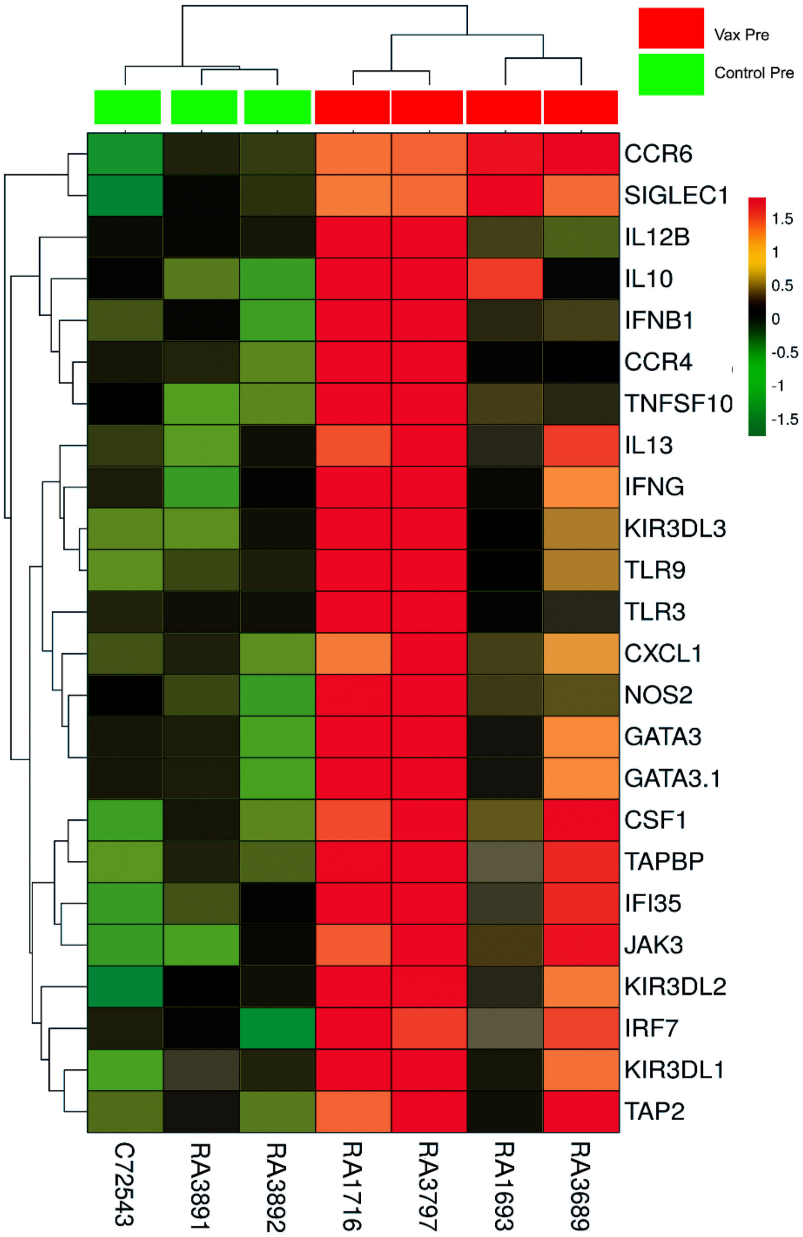

Supplemental Data, Figure S13 Legend. Hierarchical clustering of adjuvant-related transcript expression in BAL samples collected from control and vaccinated macaques prior to virus challenge (indicated as Control Pre and Vax Pre). Gene expression analysis identified differentially regulated genes in BAL samples obtained on Day -1 from control animals and Day -7 from vaccinated macaques. Heatmap shows significant (p <0.05) differential expression of a series of genes that had previously been identified (see main text) as being regulated by adjuvants alone. Up-regulated (red); down-regulated (green). Macaque RA 3830 was not sampled on day - 1.

Figure S14

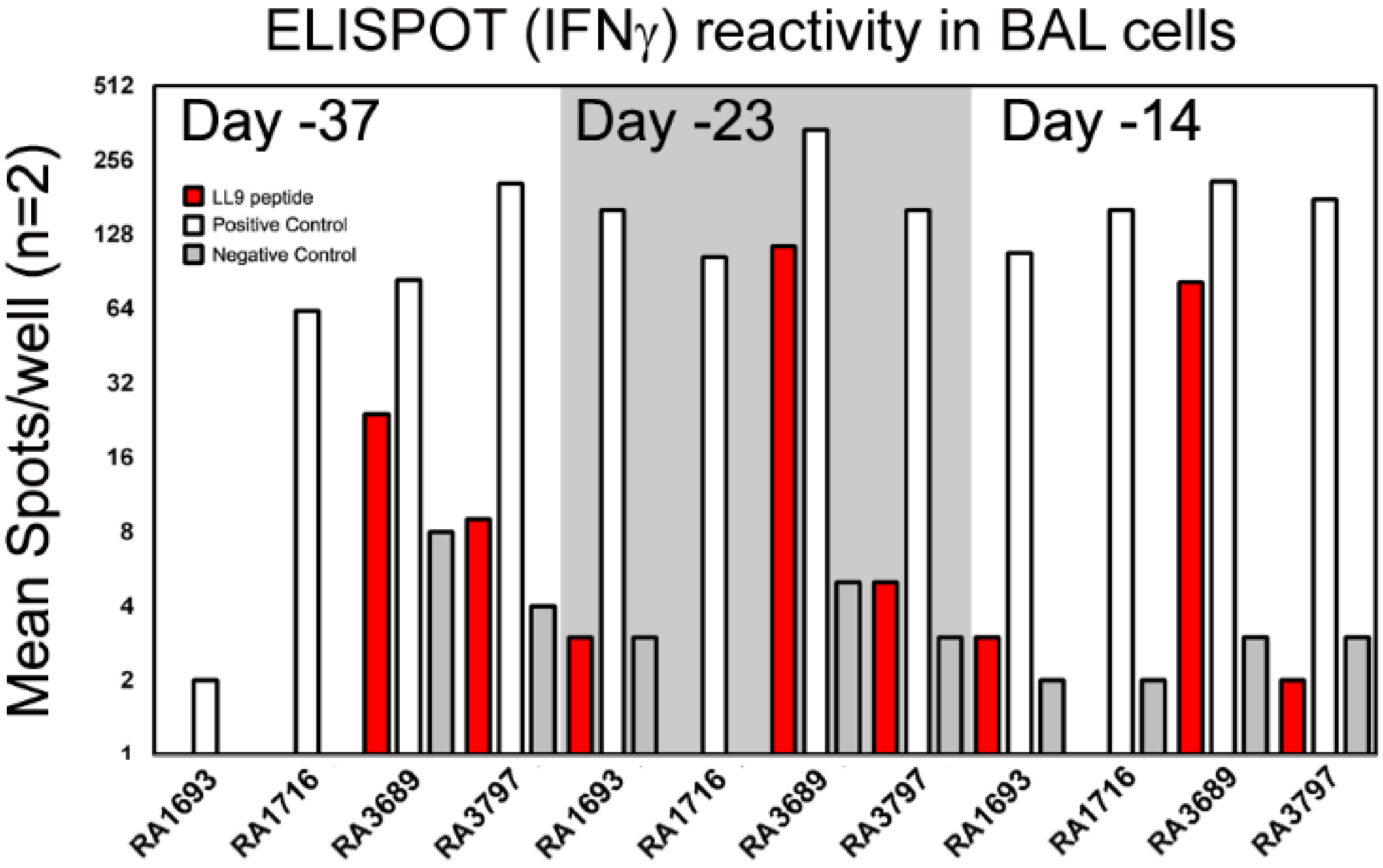

Supplemental Data, Figure S14 Legend. Immunoreactivity of BAL-associated cells from vaccinated macaques to immunizing peptides prior to SARS-CoV-2 challenge. Dates shown are assay date rather than sampling date. Concanavlin A was used as a positive control.

Supplemental data –Table S1A and S1B

**Supplemental Table S1A**											
**CTL epitope HLA coverage**	HLA allele (genotypic frequency % world)										Aggregate HLA Class I Coverage %
	HLA A*01:01 (10.09)	HLA A*02:01 (24.39)	HLA A*03:01 (9.77)	HLA A*11:01 (8.99)	HLA A*23:01 (3.06)	HLA A*24:02 (12.59)	HLA A*29:02 (2.18)	HLA A*30:02 (1.36)	HLA A*31:01 (3.02)	HLA A*68:01 (3.29)	
Epitope											
LSPRWYFYY	+[1]	-	-	+	+	+	+	+	+	-	60.51
LLLDRLNQL	-	+	-	-	-	-	-	-	-	-	39.08
KTFPPTEPK	-	-	+	+	-	-	-	-	+	+	40.03
GMSRIGMEV	-	+	-	-	-	-	-	-	-	-	39.08
ASAFFGMSR	-	-	+	+	-	-	-	-	+	+	40.03
QQQGQTVTK	-	-	-	+	-	-	-	-	+	-	
Epitope Count	1	2	2	4	1	1	1	1	4	2	91.51[2]

Notes: 1. + Indicates positive in-vitro assays for MHC binding and/or T-cell recognition [36]. 2. Calculated as previously described[78]

**Supplemental Table S1B**							
**Rhesus Mamu MHC Class I coverage – predicted binding**							
NHP Subject	R1693[1]	R1716		R3689		R3797	
Epitope							
LSPRWYFYY		*A7*01:03*				*A1*002:02*	B*068:01(WB)
LLLDRLNQL	*A1*026:01*	B:086:01 (WB)		*A1*026:01*	B*056:01(WB)	B*056:01(WB)	B*068:01(WB)
				A1*004:01(WB)		A1*004:01(WB)	
KTFPPTEPK	B*013:01(SB)	*B*083:01*	B:086:01 (WB)	*B*066:01*		*B*066:01*	
GMSRIGMEV	*A1*026:01*			*A1*026:01*			
ASAFFGMSR	B*013:01(WB)	*B*083:01*		*B*066:01*		*B*066:01*	
QQQGQTVTK	B*013:01(SB)						

Notes: 1. A typical rhesus MHC haplotype may contain two or three expressed Mamu-A genes, and up to nineteen distinct Mamu-B-like loci[79], 2. Mamu MHC in *italics* are predicted to bind based on HLA homology and in-vitro analysis [40]. 3. Mamu MHC in regular font are predicted to bind based on NetMHCpan 4.1, with a weak binder (WB) at top 2% percentile rank and strong binder (SB) at top 0.5% precentile rank.
